# Low-grade Appendiceal Mucinous Neoplasm Presenting as Adnexal Mass: A Case Report

**DOI:** 10.7759/cureus.3568

**Published:** 2018-11-10

**Authors:** Eirini V Pantiora, Dimitrios Massaras, John Koutalas, Anastasia Bagiasta, Elissaios A Kontis, Georgios P Fragulidis

**Affiliations:** 1 Surgery, Aretaieio Hospital, National and Kapodistrian University of Athens School of Medicine, Athens, GRC; 2 Anesthesiology, Aretaieio Hospital, National and Kapodistrian University of Athens School of Medicine, Athens, GRC

**Keywords:** appendices mucocele, adnexal mass, surgery, diagnosis, tumor pathology, treatment, hipec

## Abstract

An appendiceal mucocele is a dilatation of the appendix and it is the result of benign or malignant diseases, which cause the obstruction of the appendix and the consequent accumulation of mucus secretion. The preoperative diagnosis is difficult due to non-specific clinical manifestations of the disease. We present a case of an 83-year-old female patient with a history of breast cancer that was referred to our hospital for an evaluation of a right adnexal mass discovered during her yearly follow-up. The patient underwent an exploratory laparotomy with a provisional diagnosis of a right adnexal mass. A perioperative, appendiceal mucocele was diagnosed. She underwent a formal appendectomy and histopathology of the specimen revealed a low-grade mucinous neoplasm. Appendiceal mucinous neoplasms represent a rare form of pathology among all appendectomy specimens. A preoperative diagnosis is difficult due to the lack of specific symptoms and it is often misdiagnosed as an adnexal mass. The perforation of the appendix and subsequent extravasation of its contents into the abdominal cavity may lead to pseudomyxoma peritonei, which has a very poor prognosis if not treated properly.

## Introduction

A mucocele of the appendix is a rare entity contributing to 0.2%-0.7% of appendiceal pathologies and is more frequent among individuals aged 50 years or more [1-2]. Rokitansky first described it in 1842 as a dilatation of the appendiceal lumen by an abnormal accumulation of mucus [3]. The mucus material contains epithelial adenoma cells with a low or high grade of dysplasia. The rupture of the appendix may lead to the dissemination of the epithelium that produces mucins in the abdominal cavity causing mucinous ascites or pseudomyxoma peritonei.

The clinical presentation varies with more than a half of them being asymptomatic. The patient may present as acute appendicitis, a nonspecific abdominal pain, or a pelvic mass. Therefore, it may rarely receive a definitive diagnosis before surgery or it may be encountered during abdominal surgery performed for another indication [4]. Early recognition and extra intraoperative precautions must be implemented to prevent iatrogenic rupture and the subsequent spilling of potentially malignant mucin-producing cells throughout the peritoneal cavity [2]. Thus, it is important for gynecologists and general surgeons to consider an appendiceal mucocele in their differential diagnosis in the case of a pelvic mass.

In this study, we present a case of a postmenopausal woman diagnosed with a pelvic mass. The patient underwent laparotomy, which revealed an appendiceal mucocele, and a formal appendectomy was performed. We also refer to the various classification systems that have been recently proposed for appendiceal mucinous neoplasia and the recent guidelines published by the Peritoneal Surface Oncology Group International (PSOGI) and American Joint Committee on Cancer (AJCC) 8th edition [5-6].

## Case presentation

**Figure 1:**
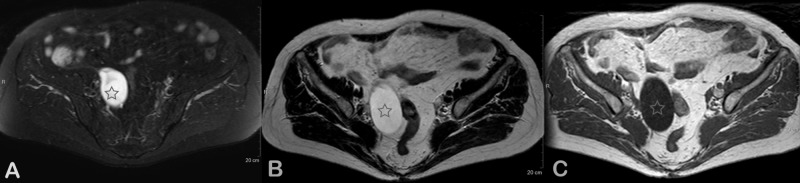
Abdominal MRI images Axial MR (A) T2W, (B) T1W, and (C) T2 SPAIR images, at the level of the pelvis minor, show a T2 hyperintense and T1 hypointense cystic lesion in the right adnexal region. No solid elements are noted. MR: magnetic resonance; T2W SPAIR: T2-weighted SPectral Attenuated Inversion Recovery

An 83-year-old female patient, with a history of breast cancer diagnosed at the age of 68, for which she underwent lumpectomy followed by radiotherapy and chemotherapy, was referred to the ob-gyn department of our hospital for an evaluation of the right adnexal mass discovered during her yearly follow-up. On physical examination, a palpable mass was found in the right hypogastric area without tenderness. Tumor markers were within normal rates, with a mild elevation of serum carcinoembryonic antigen (CEA) 5.3ng/ml (normal rates< 4.7) and of serum cancer antigen 15-3 (CA 15-3) 31.4U/ml (normal rates <28). An abdominal ultrasound showed a hypoechoic formation, sized 80.0 x 36.6 mm, below the uterus. An intravaginal ultrasound revealed a mixed texture mass, sized 8.7cm, with a solid and a cystic part in the right ovary. No free fluid was seen in the Douglas pouch. An abdominal magnetic resonance imaging (MRI) scan was carried out, which identified a cystic mass, sized 9 cm, in the right iliac fossa. The mass was in contact with the right ovary, uterus, and intestines. It was described as thin-walled without a disturbance in its molecular diffusion and with low-grade heterogeneity in its upper part, as can be seen in Figure 1.

**Figure 2:**
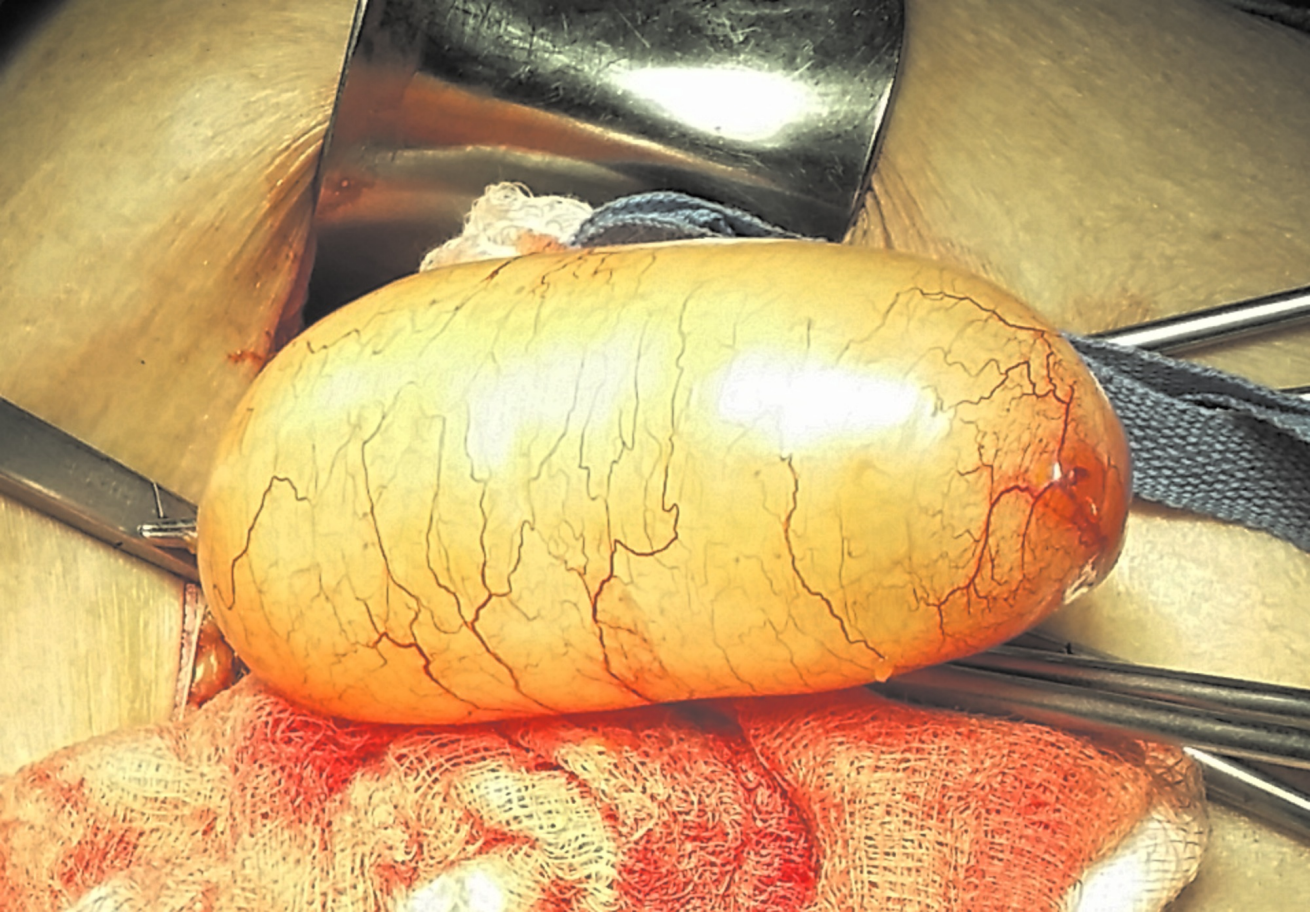
Intraoperative view of an appendiceal mucocele

The patient underwent an exploratory laparotomy under the diagnosis of a pelvic mass. The perioperative bilateral adnexa and uterus were found normal during the abdominal exploration. An appendiceal mass was revealed and a formal appendectomy was performed (Figure 2).

**Figure 3:**
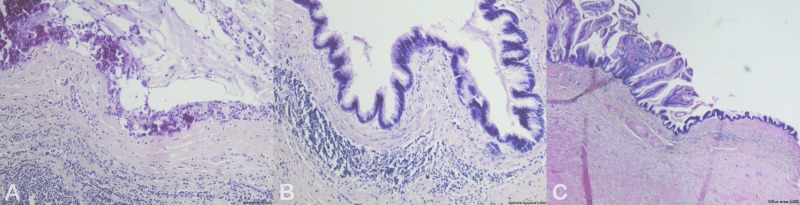
Histological images of the appendiceal specimen Histopathology of the tumor showed (A) calcifications (x100), (B) epithelial dysplasia (x100), and (C) villus area (x20)

A frozen section of the appendix specimen diagnosed cystadenoma. The postoperative course was uneventful. A pathological examination of the surgical specimen revealed a low-grade mucinous neoplasm of the appendix (Figure 3).

After a year of follow-up, the patient is asymptomatic, with no pathological imaging findings.

## Discussion

The term appendiceal mucocele refers to a dilated appendix with an increased intraluminal accumulation of mucus. It may be caused by a primary disease, which could range from an innocuous hyperplastic process (mucosal hyperplasia) to a clinically benign neoplasm (mucinous cystadenoma), ending with a truly malignant tumor (mucinous cystadenocarcinoma) [7]. The incidence of mucinous cystadenocarcinomas is 9% of appendiceal mucoceles specimens [1].

The clinical presentation is usually non-specific and the final diagnosis is intraoperative and based on the histopathological evaluation. A study done by Stocchi and coworkers found that of patients with symptoms, 27% had abdominal pain, 14% had an abdominal mass, 13% lost weight, 9% had nausea, vomiting, or both, and 8% presented with acute appendicitis [8]. It can also be presented as intestinal strangulation, appendiceal intussusception, or generalized abdominal pain [4]. Laboratory tests, such as elevated carcinoembryonic antigen (CEA) and cancer antigen (CA), are known to be associated with mucinous cystadenoma and cystadenocarcinoma but are non-specific [9]. On imaging, ultrasonography (US) may reveal the mucocele as hypoechogenic, however, echogenicity may also be seen depending on the number of acoustic interfaces (“onion skin”) caused by mucus [10]. A computed tomography (CT) examination of the abdomen is a more accurate diagnostic tool and could reveal a low-attenuated, well- encapsulated, thin-walled cystic mass in the right lower quadrant. In 50% of the cases, mural calcification was also observed and enhancing modules may suggest cystadenocarcinoma [11]. MRI could demonstrate a mass with intermediate signal intensity on T1-weighted images and homogenous T2- weighted images [12]. Hence, based on the symptoms and imaging findings, the differential diagnosis usually includes acute appendicitis, a mesenteric cyst, or an adnexal mass.

Misdraji et al. classified appendiceal mucoceles into two groups based on architectural and cytologic features: low-grade appendicular mucinous neoplasms (LAMN) characterized by villous or flat-mucinous epithelial proliferation with low degree atypia, and mucinous adenocarcinoma (MACA), characterized by tumoral invasion of the bowel wall, complex epithelial proliferation, and high-grade nuclear atypia [13]. These lesions are precursors to pseudomyxoma peritonei (PMP) that has been characterized as a localized or generalized accumulation of thick, gelatinous material in the abdominal and/ or pelvic peritoneal cavity. Most cases of PMP develop as a result of appendiceal mucinous neoplasia [14-15]. Consequently, PMP can be classified as involvement by LAMN or MACA and this distinction is of prognostic significance [13]. Some low-grade appendicular mucinous neoplasms may progress to PMP via peritoneal spread, although the amount of mucin outside the appendix is only visible by histology, leading to low-grade mucinous carcinoma peritonei. These low-grade lesions outside the lumen were labeled as LAMN type 2, whereas lesions with disease confined to the lumen were defined as LAMN type 1, as in our case [14]. According to the histology of the peritoneal disease, when LAMN type 2 is associated with a high-grade (G2) peritoneal tumor, it behaves as adenocarcinoma [13,15].

Given these unique issues with appendiceal mucinous neoplasia, the AJCC 8th edition has made significant and necessary changes to the staging of LAMN and created a new T category specifically for LAMN, termed the Tis-LAMN [6,15]. Tis-LAMN is limited to the muscularis propria, occasionally with a “pushing” pattern as a diverticulum-like growth into the muscularis. Acellular mucin extending into the muscularis propria is also classified as Tis-LAMN as long as it does not extend into the subserosa and mesoappendix or extend to involve the visceral peritoneal surface. According to the AJCC 8th edition, the pT1 and pT2 designations do not apply to LAMN because the muscularis propria is often replaced by fibrosis, making an assessment of transmural involvement by the neoplasm difficult [15]. As a result, a pT3 LAMN designation follows pTis for either acellular mucin or a diverticulum-like growth of the neoplastic mucinous epithelium into the periappendiceal adipose tissue. If acellular mucin or the neoplastic mucinous epithelium penetrates the visceral peritoneal surface, the tumor is designated as T4a. If acellular mucin or neoplastic mucinous epithelium directly invades adjacent organs or structures, the tumor is designated T4b. The Tis-LAMN diagnosis should be strictly reserved for those LAMNs that are confined to the muscularis propria after a histologic examination of the entire appendix and a diligent search by the surgeon for any evidence of disease outside of the appendix [15]. Patients with Tis-LAMN, so defined, are typically cured by complete resection of the appendix with no risk for recurrent disease [13,15-16].

For lesions with the low-grade architectural features of LAMN but with high-grade cytologic features in the absence of an infiltrative invasion within the appendix, the term high-grade appendiceal mucinous neoplasm (HAMN) was proposed by the PSOGI consensus on the diagnostic terminology of mucinous appendiceal neoplasia. The term “low-grade appendiceal mucinous neoplasm” was supported by the PSOGI consensus while the term “mucinous adenocarcinoma” should be reserved for lesions with an infiltrative invasion. It was also agreed that the term “cystadenoma” should no longer be recommended [5,15]. The AJCC 8th edition includes HAMN in the moderately differentiated (G2) grade category [6].

The treatment of an appendiceal mucocele is surgical resection. The surgical approach depends on the specific circumstances of the tumor and on clinical presentation. One of the cardinal principles in surgical management is that an intact mucocele presents no future risk for the patient. Many authors prefer the open surgical approach for the excision of a mucocele because it gives the ability for safer manipulations of the lesion. However, some authors reported that the laparoscopic and certain, minimally invasive techniques approaches are also appropriate [12,17]. In the past, all patients with appendiceal mucinous malignancy were recommended for a right colectomy. Prospective data by Gonzales-Moreno and associates showed no survival advantage with a right colectomy [7]. In the absence of peritoneal involvement, patients can be managed with appendectomy and excision of the mesoappendix. However, a diagnosis of mucinous adenocarcinoma in an appendectomy specimen should result in a subsequent right hemicolectomy to evaluate for lymph node metastases [15,17]. It should be noted that if HAMN is identified, great care should be taken to exclude the presence of associated invasive adenocarcinoma, including a comprehensive histologic evaluation of the pathology specimen [15]. In case of a ruptured appendiceal mucocele, the primary resection should be accompanied by the removal of all gross implants and any fluid or mucus must be recovered for a cytologic examination [2]. A prognosis of appendiceal mucinous neoplasm is dependent on whether the tumor progresses to PMP. In pTis-LAMN, PMP only occurs in approximately 2% whereas 20%-23% of mucinous adenocarcinomas progress to PMP [1,13]. Without this progression, the five-year survival was reported to be 32%-58% [13]. Cytoreductive surgery, chemotherapy, and hyperthermic intraperitoneal chemotherapy are suggested when the histological examination reveals the presence of PMP [18-19]. Low-grade tumors have the maximum survival benefit from these locoregional treatments [15].

The five-year overall survival for patients with disseminated low-grade (G1, well-differentiated) mucinous neoplasms ranges from 60% to 90% with an estimated 10-year overall survival of 50% [13,15]. However, the five-year and 10-year overall survival for patients with a high-grade (G3, poorly differentiated) mucinous adenocarcinoma with signet ring cells ranges from 10% to 40% and 10% to 20%, respectively [15,20].

## Conclusions

Appendiceal mucinous neoplasms represent a rare form of pathology among all appendectomy specimens. A preoperative diagnosis is difficult due to its lack of specific symptoms and signs and the final diagnosis is mainly achieved intraoperatively. It is important for gynecologists and general surgeons to consider appendiceal mucinous neoplasms in cases where an elderly woman is observed to have a mass in the right iliac fossa. Furthermore, it is important for pathologists to be aware of the recent significant changes in the diagnostic terminology, histologic criteria, and staging of appendiceal mucinous neoplasms proposed by the PSOGI and AJCC eighth edition, as they can significantly affect patient management.
